# Bioactive pedicle screws prepared by chemical and heat treatments improved biocompatibility and bone-bonding ability in canine lumbar spines

**DOI:** 10.1371/journal.pone.0196766

**Published:** 2018-05-07

**Authors:** Koji Akeda, Seiji Yamaguchi, Tomiharu Matsushita, Tadashi Kokubo, Koichiro Murata, Norihiko Takegami, Akihiko Matsumine, Akihiro Sudo

**Affiliations:** 1 Department of Orthopaedic Surgery, Mie University Graduate School of Medicine, Tsu, Japan; 2 Department of Biomedical Science, College of Life and Health Science, Chubu University, Kasugai, Japan; 3 Department of Orthopedics and Rehabilitation Medicine, Faculty of Medical Sciences, University of Fukui, Eiheiji, Japan; Kyoto Daigaku, JAPAN

## Abstract

**Background:**

Titanium (Ti)-6Al-4V alloy, which is widely used in spinal instrumentation with a pedicle screw (PS) system. However, significant clinical problems, including loosening and back-out of PSs, persist. During the last decade, a novel technology that produces bioactive Ti from chemical and heat treatments has been reported that induces the spontaneous formation of a hydroxyapatite (HA) layer on the surface of Ti materials. The purpose of this study was to study the effect of bioactivation of Ti-6Al-4V PSs on the ability of HA formation *in vitro* and its biocompatibility and bone-bonding ability *in vivo*.

**Methods:**

Ti-6V-4Al alloy PSs were prepared and bioactivated by NaOH-CaCl_2_-heat-water treatments. The HA-forming ability of bioactive PSs in simulated body fluid (SBF) was evaluated by field emission scanning electron microscopy (FE-SEM) and energy dispersive X-ray analysis (EDX). Six 11-month-old female beagle dogs were used for the *in vivo* study. Bioactive and control (without bioactivation) PSs were left and right randomly placed from L1 to L6. One and three months after surgery, lumbar spines were removed for biomechanical and histological analyses.

**Results:**

*In vitro*: The surface analysis of bioactive PSs by FE-SEM and EDX showed substantial HA deposits over the entire surface. *In vivo*: The mean extraction torque was significantly higher for bioactive PSs compared to controls PSs (P<0.01); there was no significant difference in pull-out strength between control and bioactive PSs. Histologically, the contact area between bone tissue and screw surface showed no significant trend to be greater in bioactive PSs compared to control PSs (P = 0.06).

**Conclusions:**

Bioactive PSs prepared by chemical and heat treatments formed layers of HA on the surface of screws *in vitro* that improved biocompatibility and bonding ability with bone *in vivo*. Bioactive PSs may reduce screw loosening to overcome the obstacles confronted in spinal instrumentation surgery.

## Introduction

Using spinal surgical instrumentation with a pedicle screw (PS) system has become increasingly common in spinal surgery because of its ability to achieve the rigid spinal fixation required for the treatment of degenerative spinal diseases, spinal deformity, spinal trauma and spinal tumors[1]. A PS system has recently been aggressively used for nonfusion posterior constructs, such as dynamic stabilization[2] and percutaneous PS (PPS) for spinal metastasis or infections[3, 4]. However, significant clinical problems, including loosening and back-out of PSs, persist[5–7].

Titanium (Ti) and Ti-6Al-4V alloys were originally used for industrial products. They are now widely used as endosseous implants in orthopedic surgeries because of their good mechanical properties and biocompatibility with bone tissue. To improve the safety and clinical outcome of spinal instrumentation surgeries, several strategies have been proposed for augmented PS-to-bone fixation. These include modifications in screw design and screw surfaces[8–12]. In the last decade, a novel technology using chemical and heat treatments to produce bioactivity on the surface of Ti and its alloy has been reported to induce the spontaneous formation of a surface layer of hydroxyapatite (HA) *in vivo*[13–18]. The excellent bone-bonding ability of this bioactive Ti has been shown in several animal studies[15, 19–22]. Thus, bioactive Ti technologies prepared by NaOH and heat treatments are now under clinical assessment as a prosthesis for artificial hip joints[23, 24] and porous interbody cages for lumbar interbody fusion[25].

In an effort to improve safety and clinical outcome of spinal instrumentation surgery, we applied this bioactivation technology to a PS system. The purpose of this study was to examine the effect of bioactivation of Ti-6Al-4V PSs on (1) the ability of hydroxyapatite (HA) to form *in vitro*, and (2) biocompatibility and bone-bonding ability *in vivo*.

## Materials and methods

### Preparation of bioactive pedicle screws

**Fig 1:**
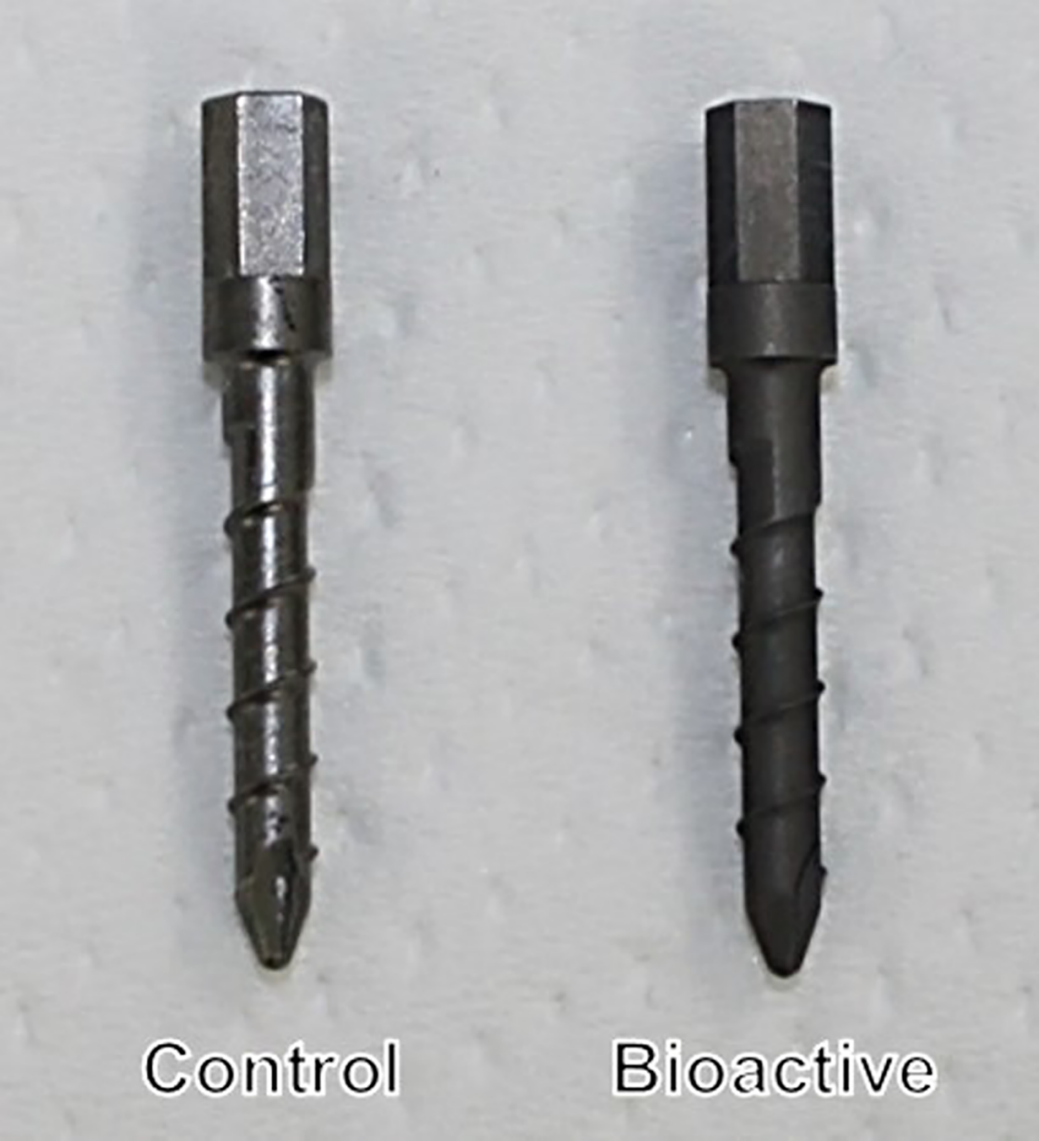
Pedicle screws (2.5 mm in diameter, 14 mm in length) were prepared from Ti-6V-4Al alloys and bioactivated by NaOH-CaCl_2_-heat-water treatments. Control: untreated pedicle screw. Bioactive: bioactive pedicle screw.

Pedicle screws (maximum diameter: 2.5 mm; minimum diameter: 2.05 mm; length: 14 mm) were prepared from Ti-6V-4Al alloys (Century Medical, Inc., Tokyo, Japan) (Fig 1). After cutting the threads, the entire screw surface was blasted using glass beads (#200). Bioactivation of PSs was performed as previously reported with some modifications[15]. Briefly, the screws were first soaked in 5 M aqueous NaOH solution at 95°C for 24 hours (alkali treatment). The screws were then soaked in 100 mM CaCl_2_ solution at 40°C for 24 hours, heated to 600°C in an electrical furnace for 1 hour, and then placed in ultrapure water at 80°C for 24 hours.

### Surface characterization of the Ti-6Al-4V alloy following bioactivation

For surface analysis, a square (10×10×1 mm^3^) Ti-6Al-4V alloy plate (Kobelco Research Institute. Inc., Japan) was prepared, and subjected to the same chemical and heat treatment for PSs described above. The surface structure was examined by field emission scanning electron microscope (FE-SEM: S-4300, Hitachi Co., Japan), thin-film X-ray diffractometer (TF-XRD: RINT-2500, Rigaku Co., Japan), and laser Raman spectrometer (FT-Raman: LabRAM300, HORIBA JOBIN YVON, France). The chemical composition and depth profile of ion distribution on plate surfaces was analyzed using Ar sputtering with X-ray photoelectron spectroscopy (XPS, PHI 5000 VersaProbe II, Ulvac, Inc., Japan).

### Evaluation of hydroxyapatite formation *in vitro*

The hydroxyapatite (HA)-forming ability of bioactive PSs was examined by incubation in simulated body fluid (SBF) as previously reported[26]. Briefly, samples were soaked in 24 ml of SBF at 36.5°C with the following ion concentrations approximating those of human blood plasma: Na^+^, 142.0; K^+^, 5.0; Ca^2+^, 2.5; Mg^2+^, 1.5; Cl^–^, 147.8; HCO_3_^–^, 4.2; HPO_4_^2-^, 1.0; and SO_4_^2-^, 0.5 mM. The SBF was prepared by dissolving reagent grade NaCl, NaHCO_3,_ KCl, K_2_HPO_4_·3H_2_O, MgCl·6H_2_O, CaCl_2_, and Na_2_SO_4_ (Nacalai Tesque Inc., Kyoto, Japan) in ultrapure water, buffered at pH = 7.4 with tris (hydroxymethyl) aminomethane (CH_2_OH)_3_CNH_2_ and 1 M HCl (Nacalai Tesque Inc., Kyoto, Japan) at 36.5°C[27]. After soaking in SBF for three days, the samples were gently rinsed with ultrapure water and dried. HA formation on the surface of screws was evaluated by FE-SEM and energy dispersive X-ray analysis (EDX; EMAX-7000, Horiba Ltd., Kyoto, Japan). HA-forming ability was also evaluated on bioactive PSs that had been removed after insertion into the pedicle of cryopreserved canine lumbar spines.

### Animal study

**Fig 2:**
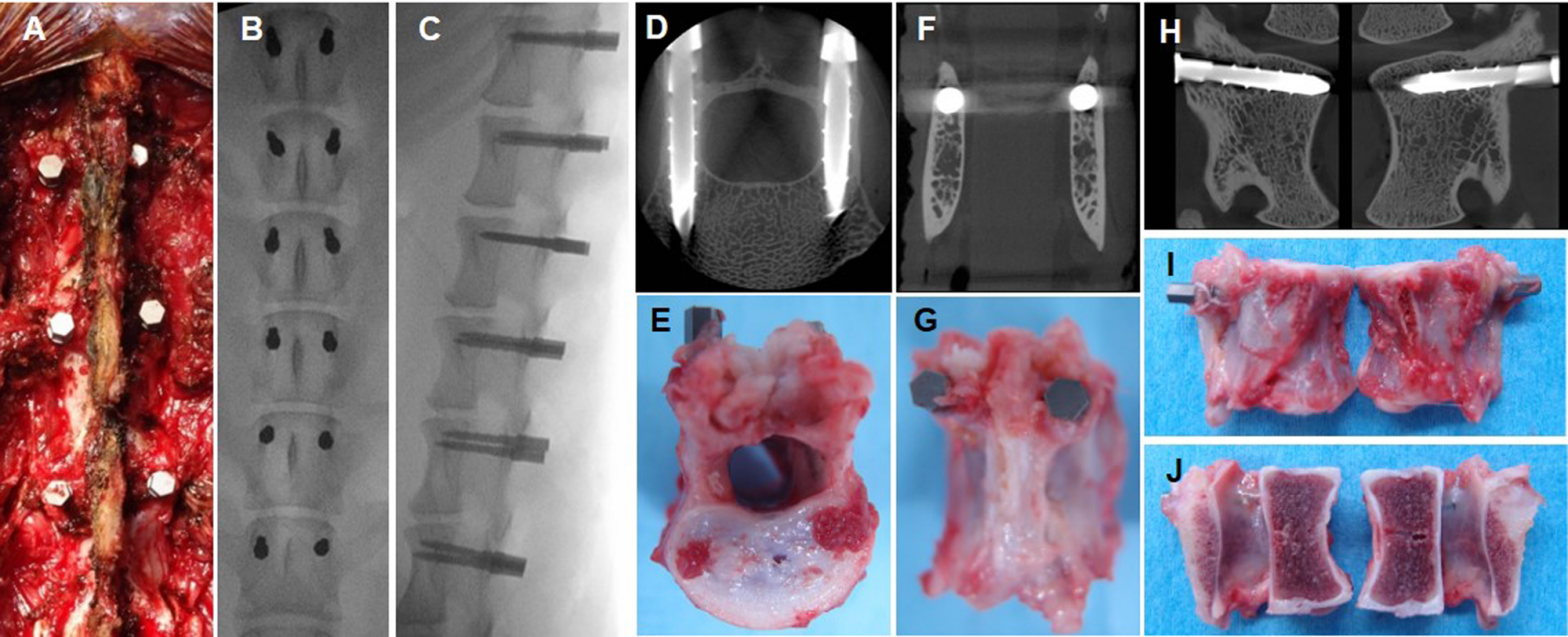
Pedicle screw insertion and harvest. A: Intraoperative picture after pedicle screw insertion. Postero-anterior view (B) and lateral (C) view of post-surgery lumbar radiograph. Representative micro X-ray computed-tomography (μCT) images (D: axial image, F: coronal image, H: sagittal image) three months post-surgery. Representative harvested specimen three months post-surgery (E: caudal view, F: posterior view, I, J: lateral view).

This animal study was carried out using the guidelines for the Care and Use of Laboratory Animals of the National Institutes of Health. The protocol was approved by the Institutional Animal Care and Use Committee of Mie University (Permit Number: 23–6). Six 11-month-old female beagle dogs (Kitayama Labes Co., Ltd., Yamatake, Japan) were used in this study. Preoperatively, the beagle dogs were anesthetized by an intravenous injection of Midazolam (0.4 mg/kg) and Propofol (4 mg/kg). Under general anesthesia with 2.0% isoflurane (Mylan Pharmaceutical Inc., Tampa, FL, USA), PSs were placed from L1 to L6 using fluoroscopy (Fig 2A–2C). The bioactive PSs were inserted randomly into the left or right pedicle at each lumbar level. Control PSs without bioactivation were inserted into the contralateral side of the pedicle. Three dogs were euthanized at one and three months after surgery. Lumbar spine removal was followed by micro X-ray computed-tomography (μCT) analysis (Fig 2D, 2F and 2H). All vertebral levels were separated at the intervertebral disc level, cut mid-sagittal and processed for histological (fixed in formalin solution) and biomechanical (stored at -30°C) analyses (Fig 2E, 2G, 2I and 2J).

### μCT analysis

Using micro X-ray CT TR_mCT2 (Rigaku, Tokyo, Japan), the samples were scanned at 50 μm resolution at 100 μA and 90 kV with a slice thickness of 0.100 mm; axial images of lumbar spines were taken (Fig 2D). Sagittal and coronal images were reconstructed using image software (One Data Viewer, J. Morita AFG. Corp., Irvine, CA USA) (Fig 2F and 2H). The accuracy of pedicle screw insertion was assessed using CT-scans and pedicle breaches were classified as previously reported with modification[28]: grade 0, the entire PS is within the pedicle; grade 1, less than 50% of the PS diameter penetrated the wall of the pedicle; grade 2, more than 50% of the PS diameter penetrated the wall of the pedicle; and grade 3, the entire PS was outside the pedicle.

**Fig 3:**
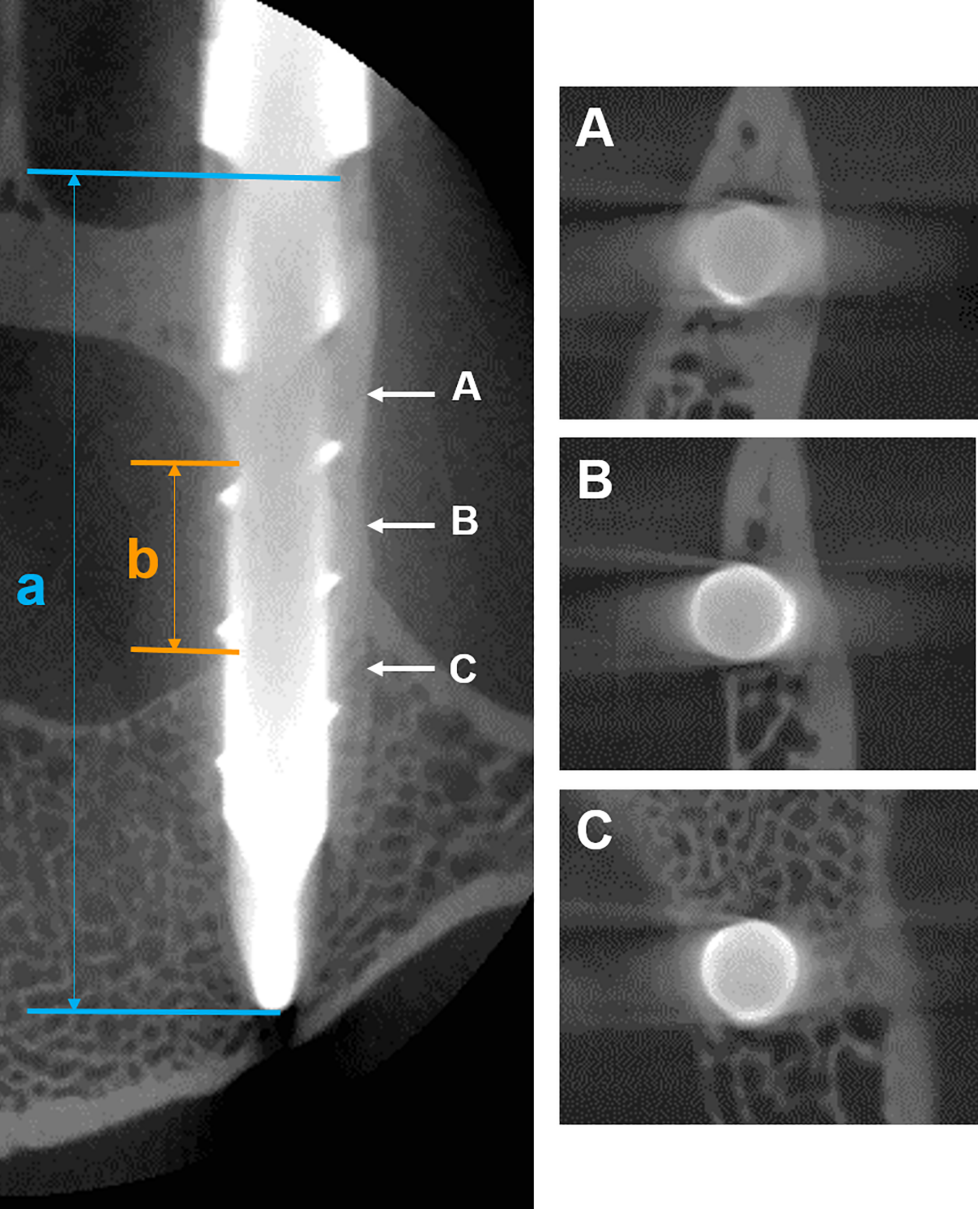
Accuracy of pedicle screw (PS) insertion.

To further evaluate the accuracy of PS insertion, the total length of the PS within the vertebra (bone tissue) (a) and the length of the pedicle breach (b) were measured using coronal CT-images (Fig 3). The ratio of PS length excluding the PS breach compared to the total PS length within the vertebra was calculated as follow: (a-b / a). The ratio of grade 0 PSs was represented as 1.

The total length of the PS within the vertebra (bone tissue) (a) and the length of the pedicle breach (b) were measured using axial CT-images (Fig 3). The ratio of PS length excluding PS breach compared to the total PS length within the vertebra was calculated as follow: (a-b / a). Three representative slices of coronal CT-images are shown (A-C). The entire screw is within the vertebra in slice A and slice C; a PS breach into the medial wall of the pedicle is found in slice B.

### Biomechanical study

A torsional screw extraction analysis was performed to evaluate the mechanical strength of the bone-implant interface. Samples (n = 35: control [n = 9] and bioactive [n = 9] PSs at 1-month post-surgery; control [n = 9] and bioactive [n = 8] PSs at 3-months post-surgery) were secured in plaster and connected to a testing machine (PT-8000, Kabaya System Machinery Co., Ltd., Tokyo, Japan). The screws were extracted at a uniform rate (1.0 rpm) for four minutes (4 revolutions). Peak and average torque (averaged data from 0 to 240 seconds, mN-mm) were determined. Stiffness was defined in the initial linear region of the torque-angular displacement curve (mN-m/degree). For evaluation of pull-out strength, specimens (n = 20: control [n = 5] and bioactive [n = 5] PSs at 1-month and 3-months post-surgery) were secured in polymethyl methacrylate (PMMA) and connected to the testing system (SERVORULSER EHF-L, Shimadzu Corporation, Kyoto, Japan). The direction of pull-movement was parallel to the long-axis of the screw. Maximum resistance against the pull-out force was calculated on the basis of the tensile curve recorded. Stiffness was defined in the initial linear region of the pull-out—displacement curve (N/mm).

Following torsional screw extraction analysis, the surface of the threaded portion of the extracted PSs was examined by FE-SEM and EDX to determine the bone-bonding ability of both the control and bioactive PSs.

### Histological analysis

One vertebra per lumbar spine (a total of 6 vertebrae) was randomly chosen from grade 0 samples and processed for histological analysis (n = 12: control [n = 3] and bioactive [n = 3] PSs at 1-month and 3-months post-surgery). The PSs with surrounding bone were then immersed in formalin solution for seven days. The samples were subsequently dehydrated with ethanol and acetone solutions. After dehydration, the samples were embedded in methyl methacrylate (MMA) resin. Samples were cut along the long axis of the screws using a saw microtome (BS-300CL, Exakt, Norderstedt, Germany). The surface of each section was further ground and polished using a micromilling system (MG-4000CS, Exakt) to a thickness of 40–60 μm. After deracination with xylene, the specimens were stained using the Villanueva-Goldner method[29].

On the threaded portion of the screw, the length of the contact area between the screw surface and bone tissues was manually measured by selecting the area of osteoid (stained red) and mineralized bone (stained green) under microscopy using image analysis software (OsteoMeasure, OsteoMetrics, Inc., Decatur, GA USA). The contact area of soft tissues, including bone marrow and fibrous tissues, was also measured. The percentage of the length of bone contact area compared to the length of screw surface was calculated. A small detachment area (gap) between the tissues and the screw surface was found in all tissue specimens. This would probably be due to the shrinkage of tissues during the process of preparing the non-decalcified specimens; the detachment area was not measured in this study.

### Statistical analysis

Biomechanical and histological data were statistically evaluated using a two-way analysis of variance (ANOVA) to evaluate differences between the groups and post-surgery period, and by the unpaired t-test to evaluate differences between the groups at each time point and between the time points of each group. The ratio of PS length excluding PS breach compared to the total PS length within the vertebra of the control and bioactive PSs was averaged and statistically analyzed using the Mann-Whitney U test. The correlation between this ratio and biomechanical data was statistically evaluated using the Spearman’s rank-order correlation test. All data are expressed as mean ± standard deviation (SD). All the statistical analyses were performed using IBM Statistical Package for Social Sciences Software (SPSS) Statistics (IBM Japan, Tokyo).

## Results

### Micro X-ray computed-tomography (μCT) analysis of pedicle screw placement

A total of 72 PSs were inserted into the pedicles of canine lumbar spines. Three screws failed to insert during surgery. μCT-image analysis revealed that 67 of the successfully inserted 69 PSs (97.1%) were adequately placed into the pedicle (grades 0 and 1) (Grade 0: 45 PSs [65.2%], grade 1: 22 PSs [31.9%], grade 2: 1 PS [1.4%], grade 3: 1 PS [1.4%]). Therefore, a total of 67 PSs were used for biomechanical and histological analyses. The average ratio of PS length excluding PS breach compared to the total PS length within the vertebra of the 67 PSs used in this study was 0.93 ± 0.11.

### Surface analysis after bioactivation treatment

TF-XRD, Raman, and XPS analyses revealed the surface layer formed by the chemical and heat treatment (bioactive surface layer) was composed of calcium titanate (CaTi_2_O_4_, CaTi_2_O_5_ and CaTi_4_O_9_), anatase, and rutile, similar to those formed on Ti metal and Ti-15Zr-4Nb-4Ta alloy subjected to the same treatments[26, 30]. The thickness of the bioactive layer measured by cross-sectional observation under FE-SEM was approximately 1.5 μm.

### *In vitro* hydroxyapatite-forming ability on the surface of bioactive pedicle screws

**Fig 4:**
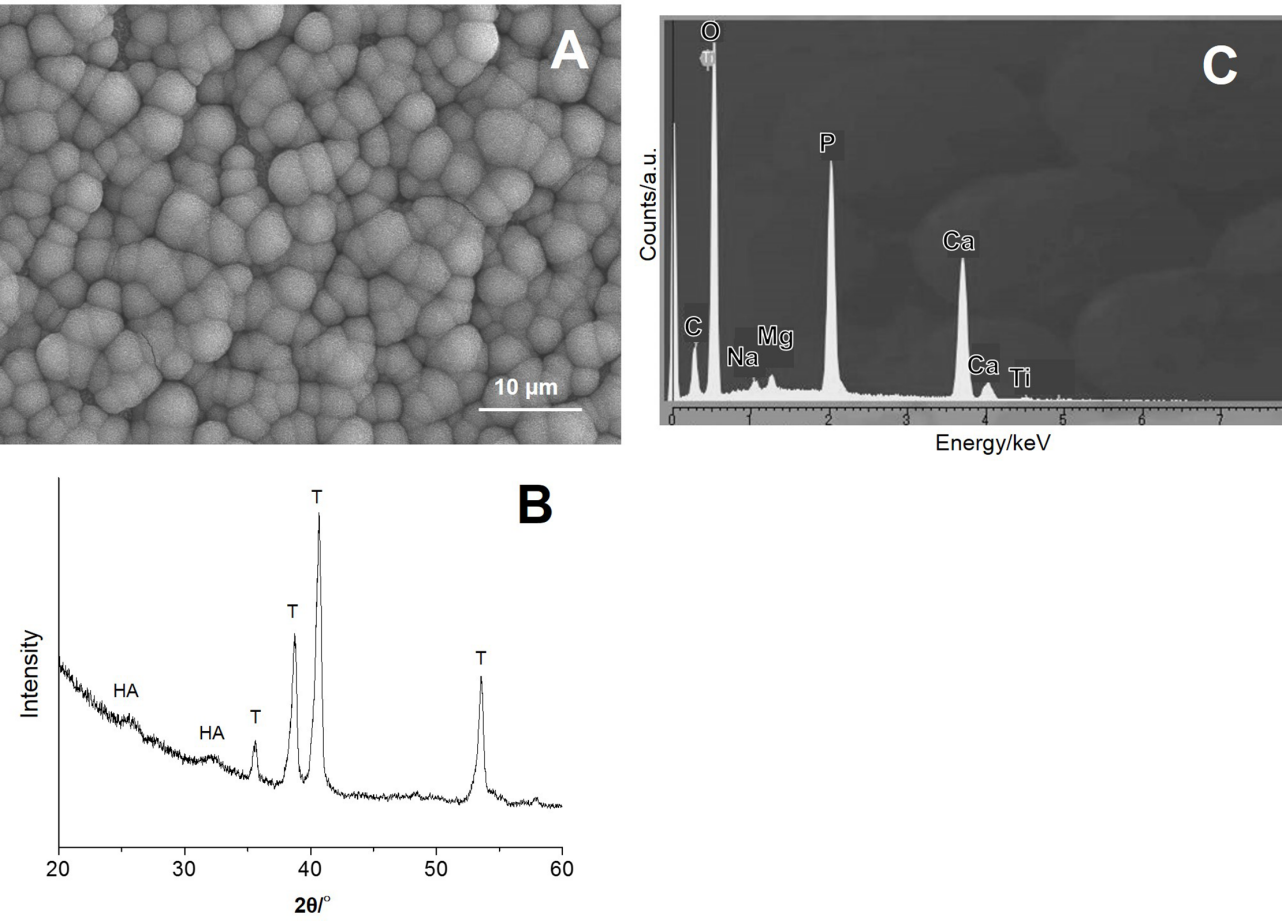
Hydroxyapatite formation (HA) on the surface of bioactive pedicle screws (PSs) after incubation in simulated body fluid (SBF) *in vitro*. A: Field emission scanning electron microscopy (FE-SEM), Bar = 10 μm. B: Thin Film (TF)-XRD pattern of the surface of bioactive PSs. T: α-Titanium (Ti). C: Energy dispersive X-ray analysis (EDX).

The surface analysis by FE-SEM showed that substantial spherical deposits covered the entire surface of bioactive PSs (n = 2) within three days after incubation in SBF (Fig 4A). In 2θ/°, TF-XRD showed broad peaks at around 26 and 32 degrees on the surface of bioactive PSs, indicating that the deposits were nano-sized crystalline hydroxyapatite (HA) (Fig 4B). The intensity of α-Ti peaks was shown to be stronger than that of HA peaks because of the sharp profile of the screw thread. EDX analysis showed that the HA deposits contained small amounts of Mg, Na and C derived from Mg^2+^, CO_3_^2-^, and Na^+^ ions in SBF (Fig 4C), in addition to Ca and P.

**Fig 5:**
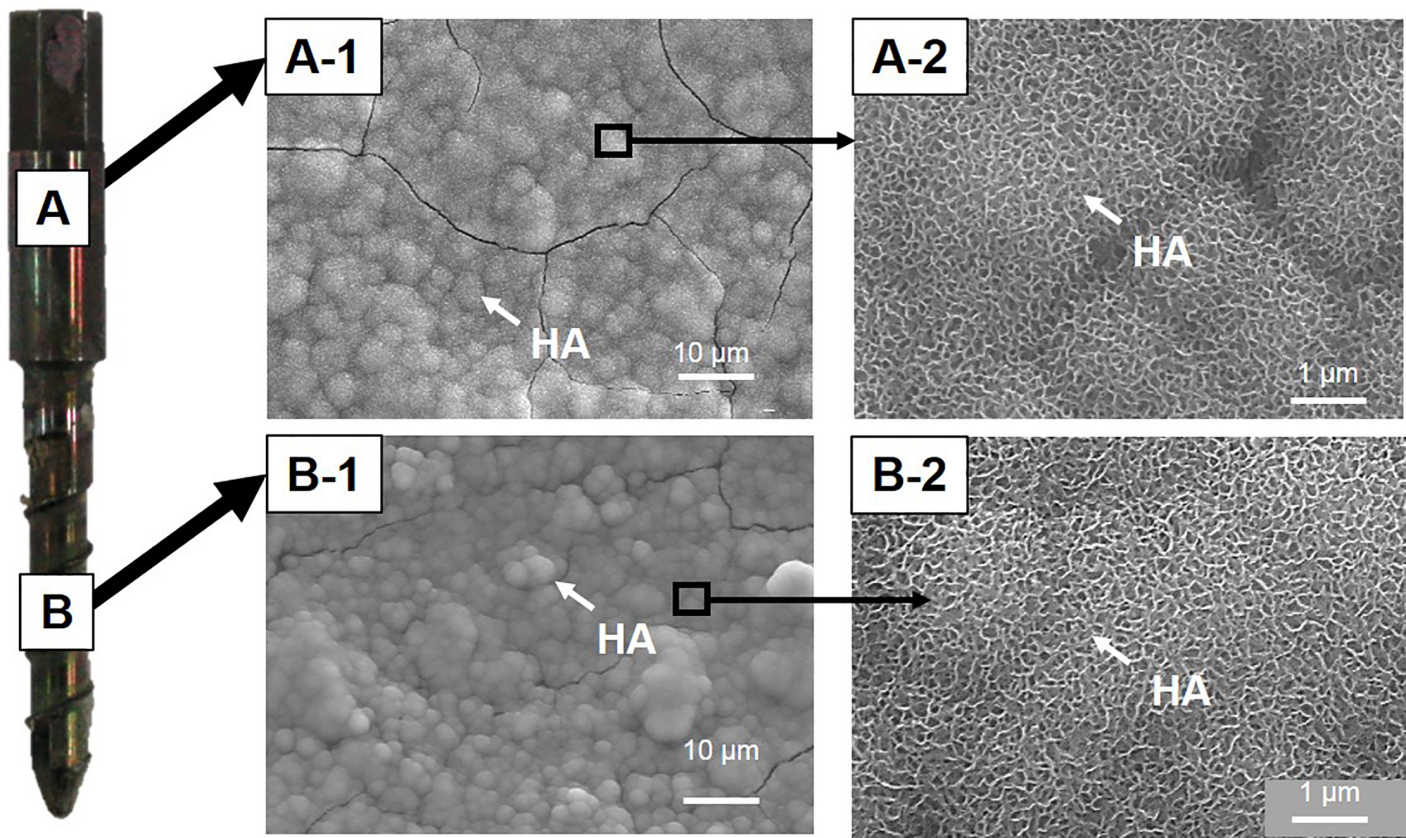
Hydroxyapatite (HA) formation on the surface of a bioactive pedicle screw (PS). Field emission scanning electron microscopy (FE-SEM) showed significant HA-formation on the surface of the PS cylindrical body (A) and threaded portion (B).

FE-SEM also showed significant HA-formation on the surface of both the cylindrical body (Fig 5A, 5A-1 and 5A-2) and threaded portion of bioactive PSs (Fig 5B, 5B-1 and 5B-2) removed after insertion into the pedicle of a cryopreserved canine lumbar spine observed by FE-SEM.

### Biomechanical analysis

#### Torsional screw extraction analysis

In the PS samples used for torsional screw extraction analysis, there were no significant differences in the ratio of PS length excluding PS breach compared to the total PS length within the vertebra between the control and bioactive PSs at both 1- and 3-months post-surgery (**1M:** control: 0.98 ± 0.05, bioactive: 1.0 ± 0.0, P = 0.44; **3M:** control: 0.93 ± 0.13, bioactive: 0.90 ± 0.15, P = 0.74). This ratio was not significantly correlated with the extraction torque (both maximum and average) at both 1- and 3-months post-surgery **(1M:** maximum: P = 0.74, average: P = 0.76; **3M:** maximum: P = 0.90, average: P = 0.75).

**Fig 6:**
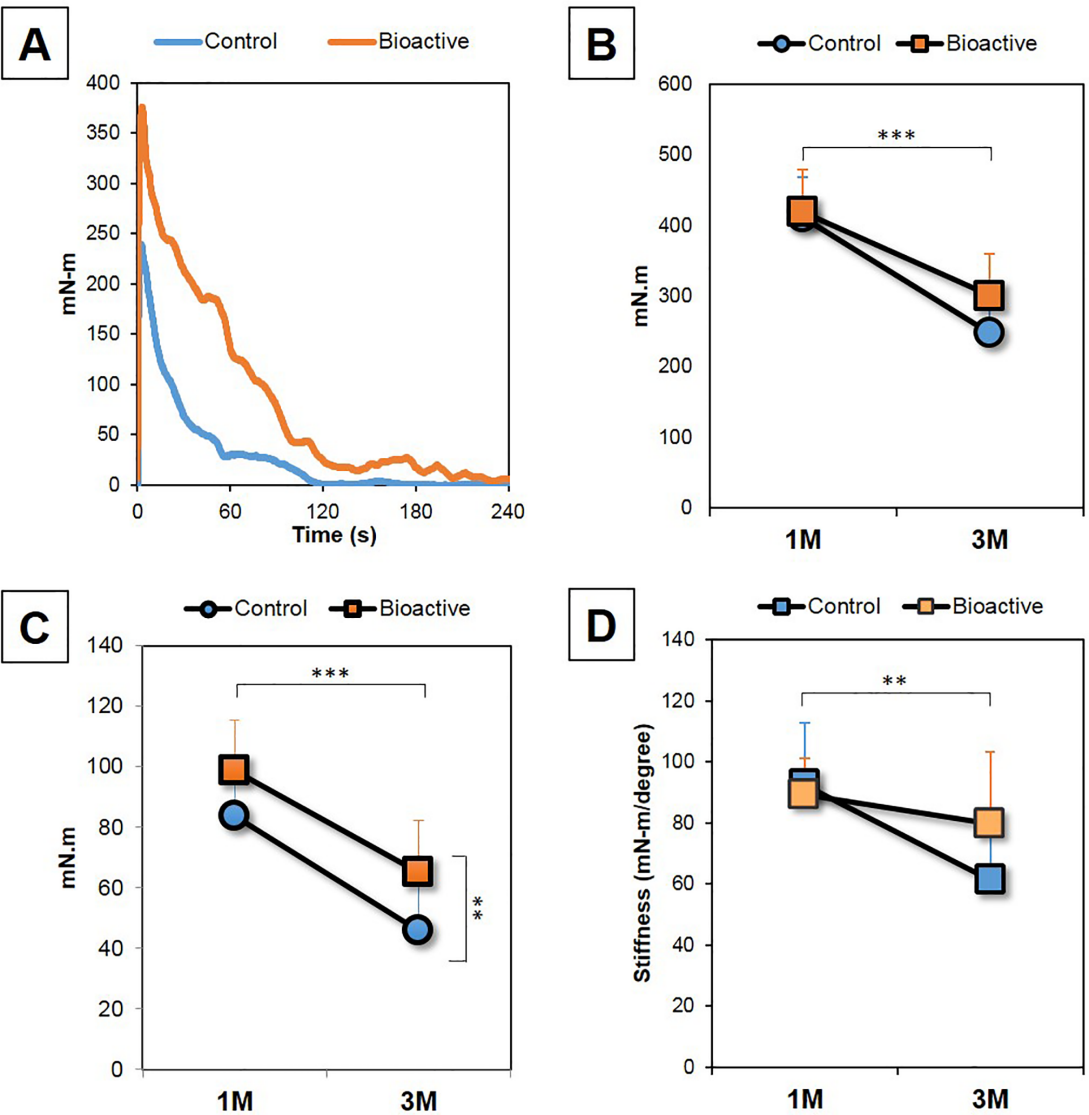
Torsional screw extraction analysis. **A:** A representative extraction torque curve at 3-months post-surgery of both an untreated (control) pedicle screw (PS) and a bioactive PS. **B.** Peak torque of untreated (control) PSs and bioactive PSs at 1- and 3- months (M) post-surgery. **C.** Average torque of the untreated (control) PSs and bioactive PSs at 1- and 3- months (M) post-surgery. **D:** Stiffness (extraction torque) of the untreated (control) PSs and bioactive PSs at 1- and 3- months (M) post-surgery. **P<0.01, ***P<0.0001, two-way ANOVA.

Representative extraction torque data from a 3-month post-surgery sample are presented in Fig 6A. The peak extraction torque was higher for bioactive PSs than control PSs. Although the torque gradually decreased for both control and bioactive PSs as the screw rotated out, the bioactive PSs maintained a higher torque throughout the recording period.

There were no significant changes in peak torque data between control (n = 16) and bioactive (n = 18) PSs (P = 0.14, two-way ANOVA, Fig 5B). When the peak torque data were analyzed at each time point, no significant differences were also identified between the groups at 1-month (1M: Control PSs [n = 9]: 411.3±57.0 mN-m, Bioactive PSs [n = 9]: 419.4±52.0 mN-m, P = 0.76) and 3-months (3M: Control PSs [n = 8]: 247.8±52.1 mN-m, Bioactive PSs [n = 9]: 300.7±69.0 mN-m, P = 0.1) post-surgery. The peak torque of both the control and bioactive PSs at 1-month post-surgery was significantly decreased at 3-months (P<0.0001, two-way ANOVA, Fig 6B). The post-hoc test also showed that there were significant differences in peak torque between 1-month and 3-months post-surgery by both control (P<0.0001) and bioactive PSs (P<0.01). On the other hand, the average rate of change from 1-month to 3-months post-surgery in the bioactive PSs (28.3% decrease) was lower than that of the control PSs (39.8% decrease).

The average torque for bioactive PSs was significantly higher than that of the control PSs (P<0.01, two-way ANOVA, Fig 6C). When the data were analyzed at each time point, no significant differences in average torque were found between the control and bioactive PSs at 1-month post-surgery (control [n = 9]: 83.5±15.1 mN-m; bioactive [n = 9]: 98.6±16.7 mN-m, P = 0.06); however the average torque of bioactive PSs was significantly higher than that of the control at 3-months (control [n = 8]: 45.9±18.3 mN-m; bioactive [n = 9]: 65.0±17.2 mN-m, P<0.05). The average torque at 1-month post-surgery of both the control and bioactive PSs decreased significantly (P<0.0001, two-way ANOVA) at 3-months (Fig 6C). The post-hoc test showed a significant decrease in average torque from 1-month to 3-months by both the control (P<0.0001) and bioactive (P<0.01) PSs, independently. However the average rate of decrease in the bioactive PSs (34.1%) was lower than that in the control PSs (45.1%).

There were no significant differences in stiffness between control PSs and bioactive PSs (P = 0.29, two-way ANOVA, Fig 6D). No significant differences in stiffness were also identified between the groups at 1-month (**1M:** Control PSs: 92.4±20.3 [mN-m/degree]; Bioactive PSs: 89.4±11.5, P = 0.70; **3M:** Control PSs: 61.5±20.5; Bioactive PSs: 79.8±23.6, P = 0.11). Stiffness of both control and bioactive PSs was significantly decreased at 3-months post-surgery compared to that at 1-month (P<0.01, two-way ANOVA, Fig 6D). The post-hoc test showed that there was a significant difference in stiffness between 1-month and 3-months post-surgery in control PSs (P<0.0001); however, no significant differences were identified in bioactive PSs (P = 0.3). The average rate of decrease for bioactive PSs (10.8%) was less than that of the control PSs (33.6%).

#### Pull-out strength analysis

In the PS samples used for pull-out strength analysis, there were no significant differences in the ratio of PS length excluding PS breach compared to the total PS length within the vertebra between the control and bioactive PSs at both 1- and 3-months post-surgery (**1M:** control [n = 5]: 0.91 ± 0.11, bioactive [n = 5]: 0.84 ± 0.10, P = 0.41; **3M:** control [n = 5]: 0.86 ± 0.01, bioactive [n = 5]: 0.84 ± 0.11, P = 0.72). There was no significant correlation between that ratio and pull-out strength at both 1- and 3-months post-surgery (**1M:** P = 0.24; **3M:** maximum: P = 0.44).

**Fig 7:**
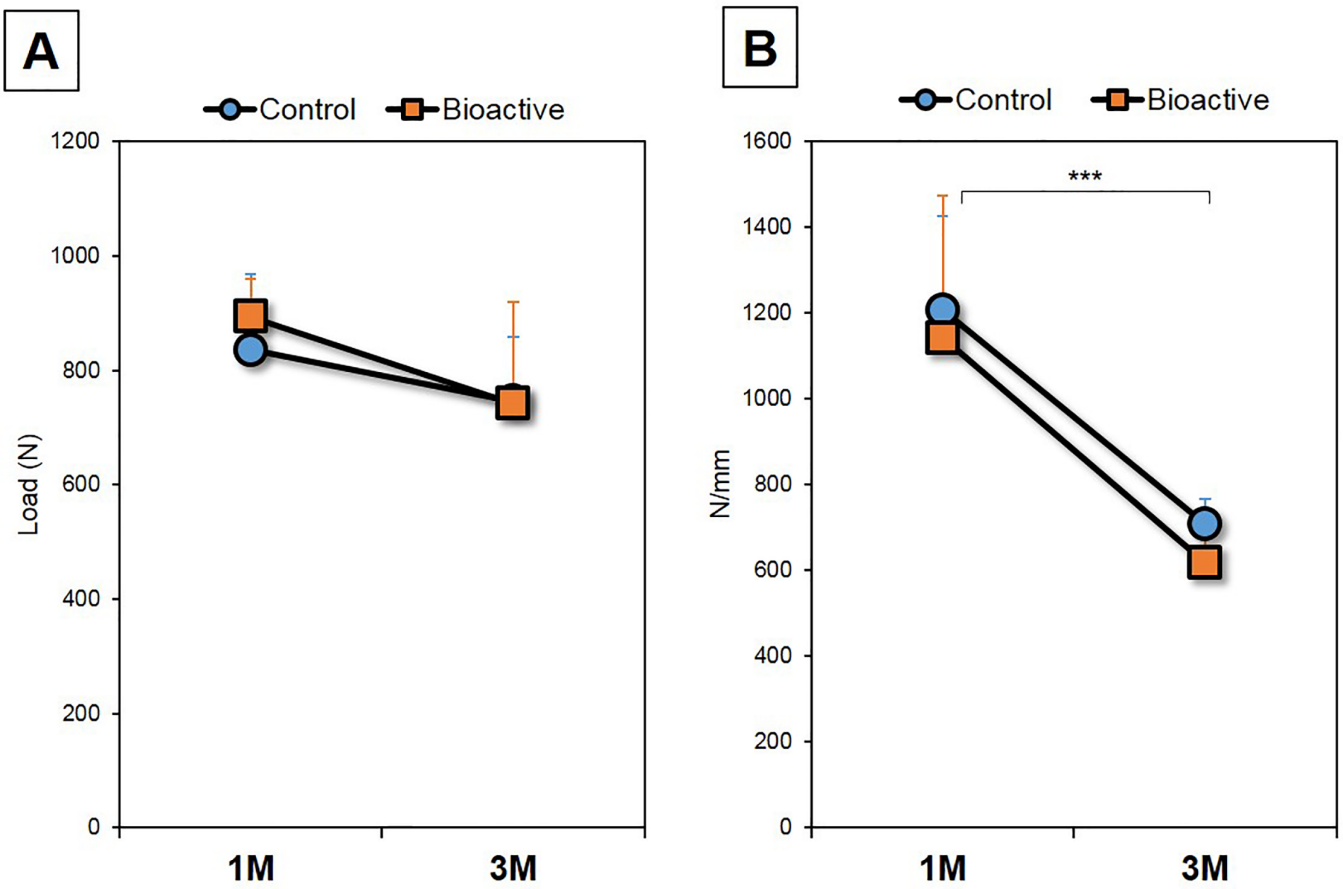
Pull-out strength. **A:** Peak load of pull-out strength of the untreated (control) pedicle screws (PSs; n = 10) and bioactive PSs (n = 10) at 1-month (1M) and 3-months (3M) post-surgery. **B:** Stiffness (pull-out strength) of the untreated (control) and bioactive pedicle screw (PSs) at 1-month (1M) and 3-months (3M) post-surgery. *** P<0.0001, two-way ANOVA.

The pull-out strength of both control and bioactive PSs did not differ significantly (P = 0.62, two-way ANOVA, Fig 7A). No significant differences in pull-out strength were identified between the groups at each time point (**1M:** P = 0.41, **3M:** P = 0.85). No significant differences in pull-out strength were found from 1-month to 3-months post-surgery (P = 0.07, two-way ANOVA, Fig 7A). No significant differences in pull-out strength were identified between each time-point for both groups (Control PSs: P = 0.27; bioactive PSs: P = 0.14).

There was no significant difference in stiffness between the control and bioactive PSs (P = 0.42, two-way ANOVA) (Fig 7B) and no significant differences were found between groups at 1-month (P = 0.41) and 3-months (P = 0.85) post-surgery. The stiffness of both the control and bioactive PSs was significantly decreased at 3-months post-surgery compared to those at 1-month (P<0.0001, two-way ANOVA) (Fig 7B). The post-hoc test showed that this significant decrease in stiffness was also identified for each group (P<0.01, respectively).

### Histological analysis

**Fig 8:**
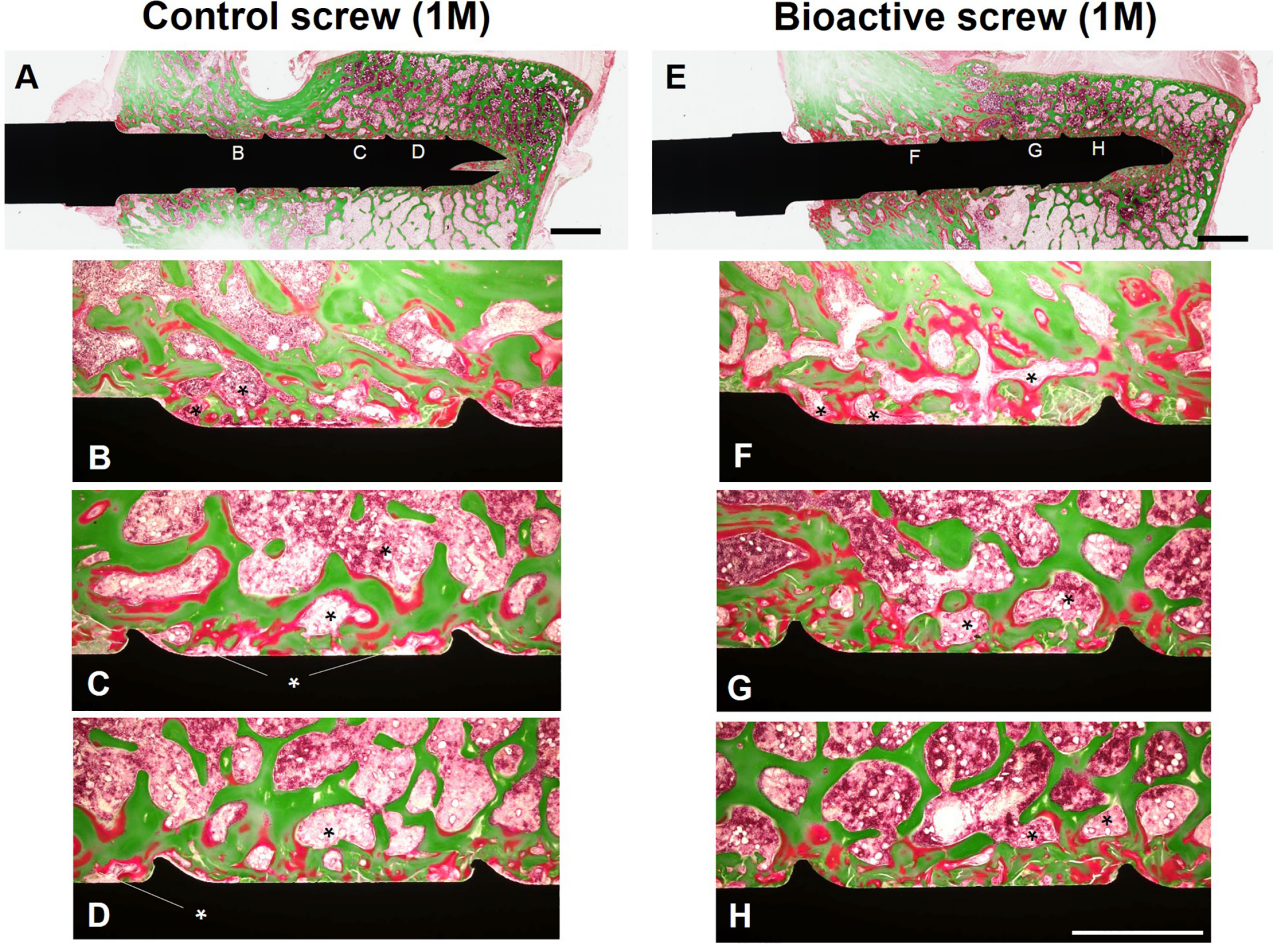
Histology at 1-month post-surgery. The specimens were embedded in methyl methacrylate (MMA) resin and stained using the Villanueva-Goldner method[29]. Control screw (A-D) and bioactive screw (E-H). Asterisk: bone marrow. Bar = 2.0 mm (A,E), Bar = 1.0 mm (B-D, F-H).

Histology at 1-month post-surgery (Fig 8) showed that the bone matrix, including osteoid (stained red) and mineralized bone (stained green), and bone marrow (asterisk) were in contact with the surface of both the control (Fig 8A–8D) and bioactive PSs (Fig 8E–8H). There were no remarkable histological changes between control (Fig 8A–8D) and bioactive PSs (Fig 8E–8H) at 1-month post-surgery. No remarkable differences in area of screw thread were found for both control (Fig 8B–8D) and bioactive PSs (Fig 8F–8H) at 1-month post-surgery.

**Fig 9:**
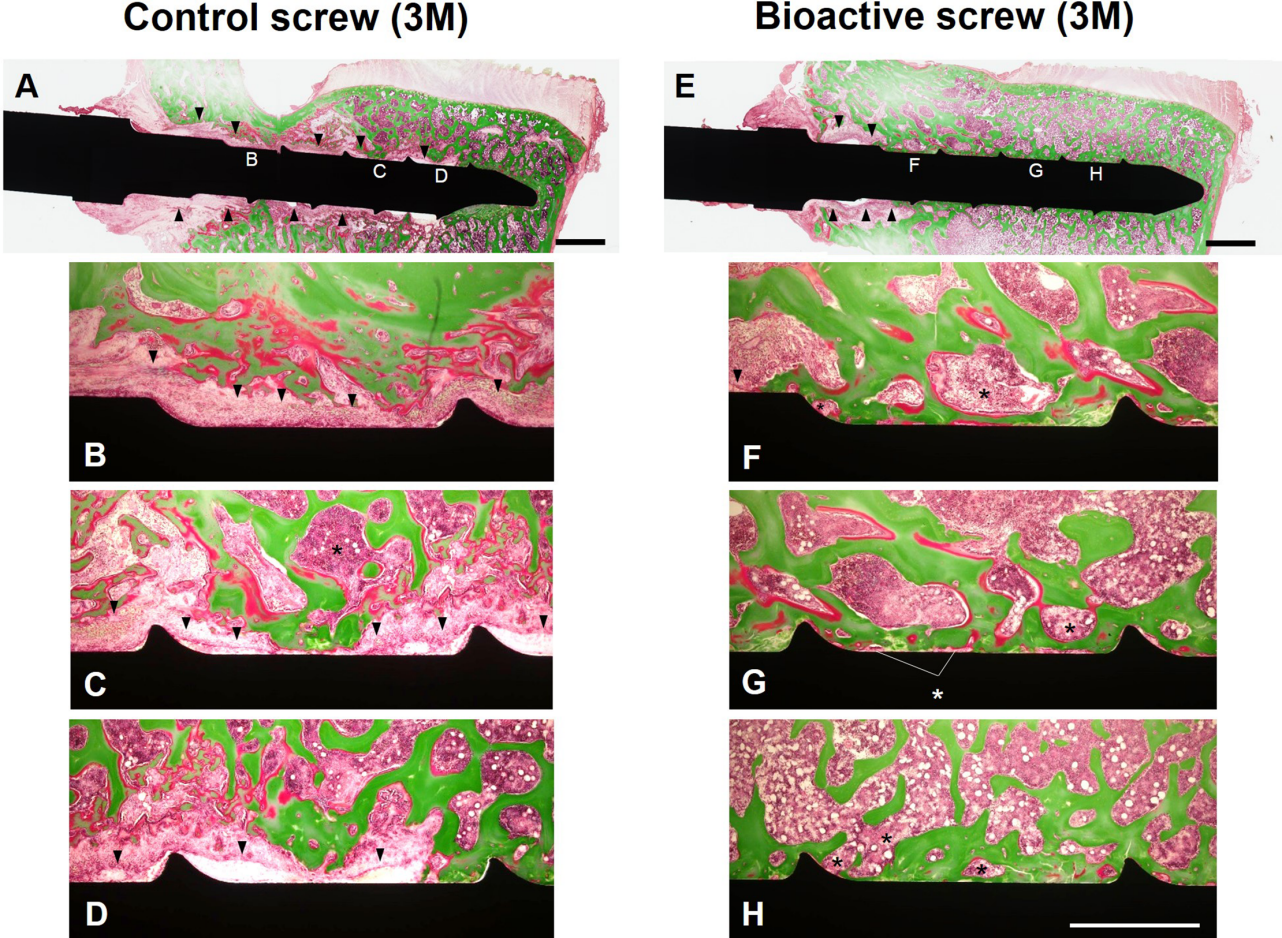
Histology at 3-months post-surgery. The specimens were embedded in methyl methacrylate (MMA) resin and stained using the Villanueva-Goldner method[29]. Control screw (A-D) and bioactive screw (E-H). Arrow heads: fibrous tissue. Asterisk: bone marrow. Bar = 2.0 mm (A,E), Bar = 1.0 mm (B-D, F-H).

At 3-months post-surgery, the area of osteoid was decreased for both control and bioactive PSs compared to those for 1-month samples. A fibrous tissue layer (arrow) was spread interstitially between the bone tissue and control group screw surface (Fig 9A–9D). On the other hand, bone matrix and bone marrow (asterisk) were closely attached to the surface of bioactive PSs (Fig 9E–9H). A fibrous tissue layer (arrow) was only found at the proximal region of the bioactive screw thread (Fig 9E).

Quantitative histological evaluations showed that the area of bone contact with the surface of bioactive PSs had no significant trend to be higher than that of the control PSs (Table 1, P = 0.06, two-way ANOVA). The soft tissue contact area of bioactive PSs was significantly lower than that of the control PSs (Table 1, P<0.05). The post-hoc test showed no significant differences in bone and soft tissue contact area between the groups when the data were analyzed at each time point (bone tissue: **1M:** P = 0.09, **3M:** P = 0.21; soft tissue: **1M:** P = 0.06, **3M:** P = 0.15). The contact area of bone tissue was significantly decreased and the contact area of soft tissue significantly increased, for both the control and bioactive PSs from 1-month to 3-months post-surgery (Table 1, bone tissue, P<0.01; soft tissue, P<0.05). For the bone tissue contact area, the post-hoc test showed no significant difference for control PSs (P = 0.07), but significant differences for bioactive PSs (P<0.05), between each time-point. No significant differences between each time-point were found in both groups for soft tissue contact area (Control PSs: P = 0.1, bioactive PSs: P = 0.27). The average rate of decrease of bone contact area of bioactive PSs (19.8%) was lower than that of control PSs (42.7%).

**Table 1:** Area of bone and soft tissues in contact with screw surface.

	1M Post-surgery			3M post-surgery**		
	Bone tissue	Soft tissue	Total	Bone tissue	Soft tissue	Total
**Control**	23.0±2.2 mm (62.2±5.5%)	13.4±2.1 mm (36.4±6.0%)	36.4±0.2 mm (98.6±1.4%)	13.6±6.1 mm (35.6±15.3%)	20.4±5.3 mm (57.8±13.1%)	33.9±1.0 mm (93.4±3.0%)
**Bioactive**	25.9±0.6 mm (70.1±2.0%)	9.9±0.9 mm* (26.8±2.3%)	35.7±1.4 mm (97.0±4.0%)	19.8±3.7 mm (56.2±7.4%)	13.3±4.3 mm* (34.6±9.4%)	33.1±1.1 mm (90.8±2.6%)

The length of contact area between bone tissue/soft tissue and screw surface of the control and bioactive pedicle screws at 1-month (1M) and 3-months (3M) post-surgery were measured. The percentages of the area of bone tissue/soft tissue in contact with the screw surface are in parentheses.

*P<0.05

**P<0.01, two-way ANOVA.

### Bone bonding ability *in vivo*

**Fig 10:**
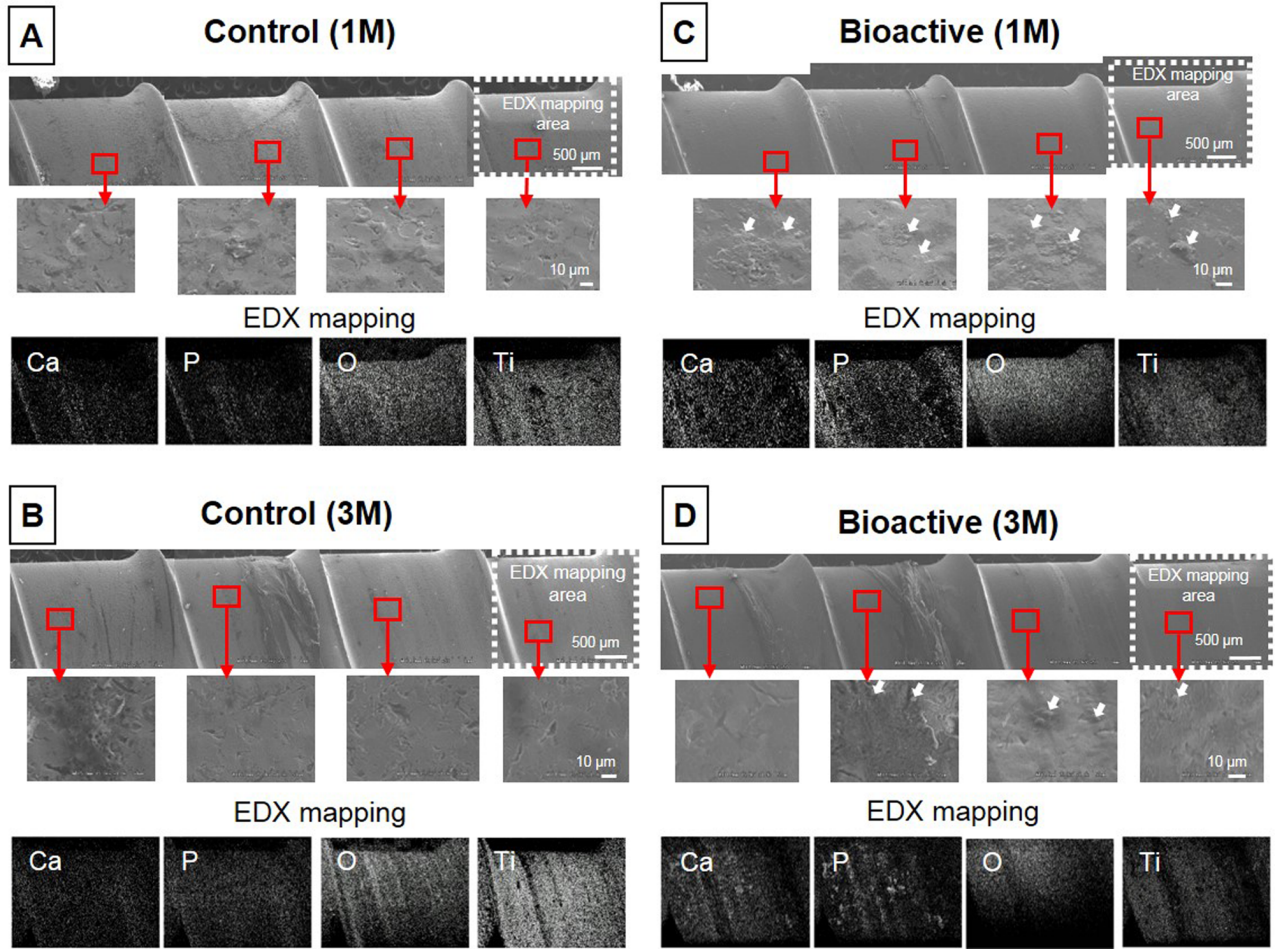
Surface analysis of retrieved pedicle screws by field emission scanning electron microscopy (FE-SEM) and energy dispersive X-ray (EDX) mapping. Following torsional screw extraction analysis, the surface of the threaded portion of removed pedicle screws (PSs) was evaluated by FE-SEM and EDX mapping. Little or no bony tissues were found on the surfaces of the control screws at 1-month (1M) **(A)** or 3-months post-surgery (3M) **(B)**. A number of bony tissues (white arrows in the magnified pictures) were observed on the surfaces of bioactive screws at 1M **(C)** and 3M **(D)** post-surgery. Scale bar: 10 μm.

Following torsion screw extraction analysis, the surface of the threaded portion of removed PSs was examined by FE-SEM and EDX (Fig 10). FE-SEM analysis showed little or no bony tissue but surfaces as blasted with glass beads were observed on the control screws implanted for 1- (Fig 10A) and 3-months post-surgery (Fig 10B). In contrast, a number of tiny bone tissues up to several μm in size (white arrows in the magnified pictures) were observed on the surfaces of bioactive screws at both implantation periods (Fig 10C and 10D). These tissues were found to be enriched in Ca and P as shown by EDX mapping (Fig 10C and 10D).

## Discussion

Pure titanium metal (Ti) and Ti-6Al-4V alloy are now widely used as biomaterials for their high biocompatibility and mechanical properties. However, these biomaterials do not always form a stable fixation with living bone for a long period of time. It has been shown that a polished Ti surface cannot bond to living bone by forming the requisite layer of thin fibrous tissue at the interface of living bone and metal[22,31]. Although a roughened Ti surface is able to come into direct contact with living bone[31], it still does not bond to it adequately.

Ti-6Al-4V alloy, as well as pure titanium metal (Ti), has been reported to bind tightly to living bone after simply being soaked in NaOH solution and subsequently heat-treated[16, 20, 22]. This bone-bonding was due to apatite formation on the surfaces of the metals *in vivo*, which was induced by the bioactive sodium titanate surface layer formed by the NaOH and heat treatment[32]. Recent studies have shown that a higher capacity for apatite formation is obtained when the Ti is soaked in CaCl_2_ solution after the NaOH treatment; this replaces the Na^+^ ions on the surface sodium titanate layer with Ca^2+^ ions through ion exchange followed by heat and water treatments[30]. This Ca and heat treatment resulted in a more stable apatite formation than that with NaOH and heat treatment alone, even after prolonged storage in a humid environment. In light of a previous series of research on bioactivation of Ti and its alloy, CaCl_2_ treatment following NaOH treatment was utilized for Ti-6V-4Al PSs in this study; this resulted in significant HA formation on the surface of bioactive PSs in SBF.

When the Ti-6Al-4V alloy was processed for NaOH-Ca-heat-hot water bioactivation treatment, a surface layer with a fine network structure approximately 1.5 μm in depth composed of calcium titanate, the bioactive layer, was identified as previously reported[26, 30]. Heat treatment following chemical treatment has induced an increased scratch resistance of more than 50 mN[30]. However surface peeling of the bioactive-layer during insertion of bioactive PSs into the vertebral pedicle has been suspected. The results of our study showed that a significant HA-forming ability on the surface of the bioactive Ti-6Al-4V PSs was retained even after removal shortly following insertion into the pedicle of canine lumbar spines. This finding indicates that the HA-forming ability of bioactive Ti-6Al-4V PSs would not be lost from surface friction during insertion of screws into bone.

In this study, the accuracy of PS insertion was quantitatively evaluated by measuring the length of PS breach into the spinal canal. No significant correlation was found between the ratio of PS length excluding PS breach compared to the total PS length within the vertebra and biomechanical data, including extraction torque and pull-out strength. As shown in Fig 3B, the thickness of the cortex bone at the pedicle is greater in the lateral wall than in the medial wall, similar to that in the human lumbar spine. Therefore, the pedicle breach (of grade 1 PSs) into the medial wall would be recognized to have little effect on biomechanical data.

The representative curve of extraction torque (as shown in Fig 6A) showed that, although the torque rapidly decreased for up to 60 seconds (one-cycle), the bioactive PSs maintained a higher torque throughout the recording period. The surface analysis of samples removed during the extraction torque analysis showed significant bony tissues attached to bioactive PSs, but not to control PSs. These results let us speculate that the extraction torque of bioactive PSs was maintained at a higher level than that of untreated PSs even after one-cycle (after peeling fracture at the surface of screw) because of the frictional resistance due to bony tissue attachment to the surface of bioactive PSs. Clinically, there is a possibility that bioactive PSs that are subject to loosening may be capable of maintaining their position or preventing further loosening that leads to the back-out of screws. Therefore, we recognize the findings from the extraction torque analysis to represent one of the important biomechanical characteristics of bioactive PSs.

The biomechanical study revealed that the extraction torque of untreated (control) PSs decreased significantly from 1- to 3-months post-surgery (peak torque: -39.5%, averaged torque: -45.6%). Similar results have also been reported from animal and clinical studies[9, 11, 33]. The reason for this mechanical change is considered to be the reduction in bone-to-screw area caused by the replacement of bone by fibrous tissue as revealed by histological analysis[11, 34–36].

The results of our histological analysis showed that no fibrous tissue was found on the surface of control and bioactive PSs at 1-month post-surgery; however a significant formation of fibrous tissues was found on the surface of control PSs of 3-months post-surgery samples. This fibrous change was identified only at the entry of the pedicle of bioactive PS samples at 3-months. These histological observations suggest that the bioactivation treatment not only induces bone formation in contact with the screw surface, but also prevents fibrous changes of bone tissues.

On the other hand, the pull-out strength analysis showed that the bioactivation of PSs induced no differences of maximum strength or stiffness compared to the control PSs throughout the observation period. Because pull-out strength is more representative of the resistance force against the bone tissue at the shared plane that connects the thread crest parts of the screw rather than at the bone-to-screw interface, bioactivation had no effect on pull-out strength in this canine animal model.

Among the surface modifications developed to add bioactivity to metal orthopedic implants, one of the accepted and commercialized bioactivation technologies is plasma-sprayed HA coating: this technique has been enthusiastically developed [37–39] and applied to PS systems[8, 9, 11, 33, 40, 41]. The effect of HA-coating on titanium-PSs was evaluated in an animal model and was shown to increase bone-bonding strength compared to untreated Ti-PSs[8, 11]. In spite of reported mechanical effectiveness, HA-coated PSs are not common in the market. Indeed, several problems are reported to remain in the clinical use of HA-coated implants including degradation and delamination of the coating[37, 42, 43]. Kawai et al.[44] recently reported a comparative study examining the effect of HA-coating and alkali heat treatments on a thermally-sprayed rough Ti surface over 16 weeks in a rabbit model. They found that HA-coating yielded significantly more bone-to-implant contact than alkali heat treatment, although alkali heat treatment produced greater bone-bonding strength than HA-coating. Thinning of the HA coat and detachment of the HA-coated layer from the implant surface were identified by SEM analysis. The authors speculated that these detrimental findings would consequently weaken the bonding ability of the HA-coated layer.

Advantages of bioactive PSs are that (1) the bioactivation does not increase the screw diameter, and (2) the HA-layer on the surface of bioactive PSs is formed within the bone tissue after screw insertion. Therefore, there is no increased insertion torque or delamination of the HA-layer during the process of screw insertion. These characteristics afford great advantages to the clinical use of bioactive PSs.

Conversely, the results of our study show that bioactivation does not completely block the time-dependent decrease of extraction torque and bone-to-screw contact area during our observation period. Therefore, at the present time in the clinical setting, bioactive PSs have the possibility of showing improvement in the initial fixation of spinal instrumentation surgery until 3-months post-surgery. To further improve the bone-bonding ability of bioactive PSs, a structural device, such as a surface modification and/or screw design to enhance the potential of bioactivation is needed.

There are several limitations that should be considered in this study. We utilized a non-fusion model with no connecting rod to the PSs. This configuration does not generate the stress transmitted to the bone-screw interface by vertebral motion. Future evaluation of a fusion model with a connecting rod and long term observation studies are needed to reflect the clinical use of bioactive PSs. Although the lumbar vertebrae (L1 to L6) were randomly selected for biomechanical and histological testing, it remains unknown whether the biomechanical properties and bone forming capacity of each canine lumbar spine could be recognized as equivalent. Therefore, it should be kept in mind, although it might be to a minor extent, the possibility that the lumbar vertebral level may affect the biocompatibility and bone bonding ability of bioactivated PSs.

## Conclusions

In this study, we have applied chemical and heat treatment bioactivation technology to conventional Ti-6Al-4V alloy PSs. This bioactive treatment induced formation of a layer of HA on the surface of Ti-6Al-4V alloy screws *in vitro*. Our canine animal study showed a significant increase in the extraction torque and bone-to-screw contact area in treated PSs, indicating this bioactivation treatment significantly increased biocompatibility and bonding ability with bone tissue *in vivo*.

The challenging application of bioactivation technology to a pedicle screw system could lead to innovative improvements in clinical outcome and safety of spinal instrumentation surgery.
